# Germline truncating mutations in both *MSH2* and *BRCA2* in a single kindred

**DOI:** 10.1038/sj.bjc.6601424

**Published:** 2004-01-20

**Authors:** I Thiffault, N Hamel, T Pal, S McVety, V A Marcus, D Farber, S Cowie, J Deschênes, W Meschino, F Odefrey, D Goldgar, T Graham, S Narod, A K Watters, E MacNamara, D Du Sart, G Chong, W D Foulkes

**Affiliations:** 1 Program in Cancer Genetics, Department of Oncology and Human Genetics, McGill University, Montreal, Quebec, Canada; 2 Department of Pathology, McGill University, Montreal, Quebec, Canada; 3 Department of Diagnostic Medicine, SMBD-Jewish General Hospital; 4 Research Institute of the McGill University Health Centre, Montreal, Quebec, Canada; 5 Centre for Research in Woman's Health, University of Toronto, Toronto, Ontario, Canada; 6 Murdoch Children's Research Institute, Melbourne, Australia; 7 Department of Genetics, North York General Hospital, University of Toronto, Toronto, Ontario, Canada; 8 IARC, Lyon, France; 9 Preventive Oncology Program, Toronto Sunnybrook Regional Cancer Centre, Toronto, Ontario, Canada

**Keywords:** *MSH2*, *BRCA2*, microsatellite instability, colorectal cancer, breast cancer, astrocytoma, multiple primary cancers

## Abstract

There has been interest in the literature in the possible existence of a gene that predisposes to both breast cancer (BC) and colorectal cancer (CRC). We describe the detailed characterisation of one kindred, MON1080, with 10 cases of BC or CRC invasive cancer among 26 first-, second- or third-degree relatives. Linkage analysis suggested that a mutation was present in *BRCA2.* DNA sequencing from III: 22 (diagnosed with lobular BC) identified a *BRCA2* exon 3 542G>T (L105X) mutation. Her sister (III: 25) had BC and endometrial cancer and carries the same mutation. Following immunohistochemical and microsatellite instability studies, mutation analysis by protein truncation test, cDNA sequencing and quantitative real-time PCR revealed a deletion of *MSH2* exon 8 in III: 25, confirming her as a double heterozygote for truncating mutations in both *BRCA2* and *MSH2*. The exon 8 deletion was identified as a 14.9 kb deletion occurring between two Alu sequences. The breakpoint lies within a sequence of 45 bp that is identical in both Alu sequences. In this large BC/CRC kindred, MON1080, disease-causing truncating mutations are present in both *MSH2* and *BRCA2.* There appeared to be no increased susceptibility to the development of colorectal tumours in *BRCA2* mutation carriers or to the development of breast tumours in *MSH2* mutation carriers. Additionally, two double heterozygotes did not appear to have a different phenotype than would be expected from the presence of a mutation in each gene alone.

*BRCA1* and *BRCA2* are the most important susceptibility genes for breast and ovarian cancer, and mutations in these two genes account for >80% of all kindreds with hereditary breast/ovarian cancer and for about 2–3% of breast cancer (BC) cases overall. Hereditary nonpolyposis colorectal cancer (HNPCC) is the most common form of hereditary colorectal cancer (CRC) (Lynch and de la Chapelle, 1999) and is responsible for 0.5–3% of all cases of CRC. Mutations in the DNA repair genes *MLH1* and *MSH2* segregate in up to 90% of HNPCC pedigrees (Peltomaki, 2001).

Several groups have studied the genetic relationship between breast and colorectal cancer, with varying findings. In one large Dutch family with a segregating *BRCA1* mutation, there are several mutation carriers who have developed CRC. However, detailed analysis suggested that the *BRCA1* mutation was not contributing to the CRCs observed. By contrast, in a large international study, Thompson *et al* (2002) observed a relative risk of 2.03 (*P*<0.001) for CRC in *BRCA1* mutation carriers, compared with general population cancer incidence. Studies in HNPCC kindreds have shown no excess of BC, or have shown that when BC does occur, microsatellite instability (MSI), a hallmark of HNPCC-related cancer, is usually absent (Aarnio *et al*, 1995; Caluseriu *et al*, 2001). Interestingly, Borg *et al* reported a family with two missense mutations in *MLH1* and a single truncating mutation in *BRCA1.* The double heterozygote for the missense *MLH1* and truncating *BRCA1* mutations developed BC but not colorectal or endometrial cancer. The authors proposed a possible protective effect of *BRCAI* deficiency against carcinogenic event in the colon (Borg *et al*, 2000).

In general, studies of female BC in HNPCC families have found little evidence for MSI (Muller *et al*, 2002), but Boyd and colleagues did find a role for MMR genes in BC susceptibility by performing molecular genetics studies in five patients with BC from HNPCC families. Microsatellite instability was observed in three of the five tumours. In one family member with a 4-bp frame-shift mutation in *MLH1*, expression of only the mutant allele was observed in the BC tissue (Risinger *et al*, 1996).

It is not uncommon to see pedigrees with several cases of BC and CRC. Given this clinical observation, and the data presented above, the question of whether a single gene that predisposes to both BC and CRC exists is pertinent. Here, we describe the investigation of one of the most convincing BC/CRC pedigrees reported. MON1080 is a 26-member kindred that includes five cases of CRC and five cases of BC (Figure 1

**Figure 1:**
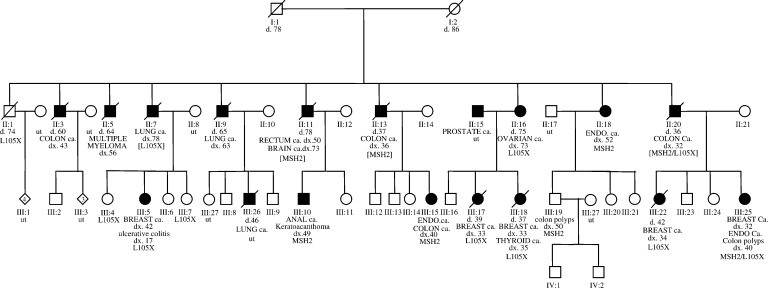
Hereditary breast and CRC Family MON1080. The numbers immediately under the symbols are individual numbers. The abbreviation ‘ut’ indicates untested. Mutations in brackets are inferred. MSH2=*MSH2* exon 8 deletion present. L105X=BRCA2^*^L105X mutation present. ENDO=endometrial cancer.

). The family meets Amsterdam criteria I for the diagnosis of HNPCC (Vasen *et al*, 1999), and nine of the 10 cases of cancer were diagnosed under the age of 45 years.

## PATIENTS AND METHODS

### Patients

A detailed family history was taken from a total of 26 related individuals from kindred MON1080. There were 17 female and nine male subjects. The details are shown in Figure 1, and all cancers were histopathologically confirmed, unless otherwise indicated.

### Immunohistochemistry (IHC) analysis

Immunohistochemistry for MLH1 and MSH2 proteins was performed as a screening test for DNA mismatch repair-deficient tumours. Mouse monoclonal antibodies G168-728 (*MLH1*, BD Pharmingen, San Diego, CA, USA) and FE11 (*MSH2*, Oncogene Research Products, San Diego, CA, USA) were used (Marcus *et al*, 1999). Surrounding normal mucosa and lymphocytes served as internal positive controls. MLH1 and MSH2 IHC was performed on the villoglandular polyp and the endometrioid adenocarcinoma of the uterus from patient III: 25; on the anal carcinoma and keratoacanthoma of patient III: 10; and on the astrocytoma of patient II: 11. Immunohistochemistry was also performed on the available BC from patients III: 5, III: 18, III: 17, III: 22 and III: 25 for ER (clone 6F11, Ventana, Tucson, AZ, USA), PR (clone 16, Zymed, San Francisco, CA, USA), CDH-1 (clone 4A2 C7, Zymed, San Francisco, CA, USA), MIB-1 (clone MMI, Novocastra, Newcastel, UK), P53 (clone DO-7, Dako, Denmark) and HER2 (clone Tab 250, Zymed, San Francisco, CA, USA) proteins.

### Microsatellite instability analysis

Microsatellite instability analysis was performed on microdissected tissues using five MSI markers: BAT25, BAT26, D2S123, D5S346 and D17S250. According to international criteria, MSI is defined as high-degree instability (MSI-H) if there is instability at two out of five loci (Boland *et al*, 1998). Microsatellite instability was performed on DNA extracted from the villoglandular polyp and the endometrial cancer of patient III: 25, on the anal carcinoma and keratoacanthoma of patient III: 10 and on the astrocytoma of patient II: 11. Following extraction of DNA from paraffin-embedded tissue, PCRs were performed using primer pairs, one of which was fluorescently labelled with Cy5.5. The products were visualised by fragment size analysis on an OpenGene System (Visible Genetics, Ontario, Canada). Anal carcinoma is not a common tumour in HNPCC kindreds, but we tested the anal and skin tumour of patient III: 10 because skin tumours are a feature of the HNPCC variant, Muir–Torre syndrome (MTS) and keratoacanthoma with MSI-H and loss of expression of MMR genes (*MSH2* or *MLH1*) have been reported to occur in MTS (Honchel *et al*, 1994; Kruse *et al*, 1996; Entius *et al*, 2000).

### DNA and RNA extraction and RT–PCR

Peripheral blood lymphocytes were separated from 16 ml of whole blood using the Accuspin™ System-Histopaque®-1077 from Sigma Diagnostics (St Louis, MO, USA). Total RNA was extracted using Trizol Reagent (Invitrogen Canada, Burlington, Ontario, Canada). DNA was extracted with the PUREGENE® Genomic DNA Isolation Kit from Gentra Systems (Minneapolis, MN, USA). Oligo(dT)-primed cDNAs were synthesised using the SuperScript™ Preamplification System for First Strand cDNA Synthesis kit (Invitrogen). The complete open reading frame of *MSH2* was then amplified as two overlapping segments from 2 *μ*l of the cDNA reaction using primers previously described by Luce *et al* (1995).

### Protein truncation test (PTT) and sequence analysis of *MSH2* cDNA

We used the TNT® Quick-Coupled Transcription/Translation kit from Promega (Madison, WI, USA) for PTT. The PCR-amplified cDNA segments were transcribed and translated *in vitro* in a reaction mixture containing ^35^S-labelled methionine, and the resultant polypeptides were separated on a 12% polyacrylamide SDS gel, dried and subjected to autoradiography. Sequencing was carried out on PCR-amplified cDNA segments by cycle sequencing using Thermo Sequenase enzyme. PCR products were separated by electrophoresis on low-melt agarose gel. The fragments were excised from the gel and purified using the QIAquick Gel Extraction kit (QIAGEN, Mississauga, Ontario, Canada). The purified fragments were then sequenced using the Thermo Sequenase Cy5.5 Dye Terminator Sequencing Kit from Pharmacia (Uppsala, Sweden).

### *MSH2* mutation analysis by real-time PCR

The real-time PCR was carried out as a duplex PCR amplifying exon 8 MSH2 and CFTR exon 24 as an internal control in a reaction volume of 25 *μ*l using the Brilliant QPCR kit (Stratagene, Integrated Sciences, East Kew Victoria, Australia). TaqMan fluorescent probes were synthesised according to the Applied Biosystems primer express software program, allowing standard amplification thermal cycling conditions (denaturation 95°C for 10 min, and subsequent cycles of 95°C for 15 s and 60°C for 1 min for 40 cycles). The PCR reaction mixture consisted of 200 nM each of MSH2 exon 8-specific forward 5′-AGAAATTATTGTTGGCAGTTTTTGTG-3′ and reverse 5′-CATATCTAAAGTTGTTTCTATCATTTCCTG-3′ primers and the CFTR forward 5′-GAAGAGAACAAAGTGCGGCAG-3′ and reverse 5′-TTGCCGGAAGAGGCTCCT-3′ primers, 100 nM FAM-labelled MSH2 exon 8-specific probe (6FAM-CTCCTCTTACTGATCTTCGTTCTGACTTCTCCAAG) and 400 nM Hex-labelled CFTR exon 24 probe (HEX-ACGATTCCATCCAGAAACTGCTGAACGA), to a final concentration of 5.0 mM MgCl_2_ and 200 ng DNA sample. Amplifications were carried out in the iCycler detection system (Biorad, Regents Park, NSW, Australia) and analyses were performed in triplicate.

### *MSH2* genomic DNA sequencing

The region containing the deletion was first amplified from genomic DNA by long-range PCR using the Expand LT and Expand 20Kb^PLUS^ PCR Systems (Roche, Indianapolis, IN, USA) and the following primers: MSH2 IVS7-5K-F Tm 68 (5′-ACTTCTTACTCCTTACTTCCTACTT-3′), MSH2 IVS8-10K-R Tm 68 (5′-AACAGGAGAGACCGCTAATAGATA-3′), TAAA-Rpt (5′-TGAGTATTGCTCTCTTGCTATCTTG-3′), and H2 EX8 ALUYF (5′-AACTTTGCCACCCATTTCAG-3′). The deletion region was also amplified using Platinum Taq (Invitrogen), the PCR Core Kit (Qiagen), and the following primers: H2 EX8 ALUYF (5′-AACTTTGCCACCCATTTCAG-3′) and MSH2, IVS8 10318R (5′-TTTGCTTGCTGATGTTCTGG-3′). The reaction mixture was prepared according to the manufacturer's protocol. The following thermocycling conditions were used: denaturation 95°C for 2 min, and 35 cycles of 95°C for 10 s, 60°C for 30 s and 68°C for 20–30 min, depending on the expected size of the wild-type product. The PCR products that contained the expected deletion were cut from the agarose gel, purified by QIAquick PCR purification Kit (Quiagen, Chatsworth, CA, USA) and sequenced using the same primers and the Thermo Sequenase Cy 5.5 Terminator Cycle Sequencing Kit (Pharmacia) according to the manufacturer's instructions.

### *BRCA2* mutation analysis

DNA from individual III: 22 was sent to Myriad Genetics Laboratories for a complete sequencing analysis of *BRCA1* and *BRCA2*. Following this, we used direct sequencing for individuals III: 22 and III: 25. Other family members were subsequently tested by single-strand conformation analysis (SSCA) using primers flanking the mutation: F-5′-CCGCTGTACCAATCTCCTGT-3′ and R-5′-GAGACTGATTTGCCCAGCAT-3′. PCR products were amplified using the Taq Gold amplification system (Perkin-Elmer) in the presence of [^35^S]dATP at 95°C (30 s), 60°C (30 s), 72°C (30 s) for 35 cycles, then separated on a 0.5 × MDE gel for 6 h 30 min (25 W, 4°C). The presence of an additional band was evidence for the presence of the mutation.

### Haplotype analysis

Haplotype analysis was performed on each individual using single-nucleotide polymorphisms (SNPs) and microsatellites within or near *BRCA2* or *MSH2*. Two tetranucleotide repeat microsatellites (TTTA: intron 2; TAAA: intron 7) within *MSH2* on chromosome 2 (Desai *et al*, 2000) were visualised by fragment size analysis on an OpenGene System (Visible Genetics, Ontario, Canada). Five polymorphic markers within or near *BRCA2* (D13S1694, D13S1695, D13S1697, D13S260 and D13S1698) were used to distinguish between the chromosomes segregating in the family (Table 1

**Table 1:** Mutation and haplotype results in family MON1080

**Generation/name**					***BRCA2* genotype**					***MSH2* genotype**		
**I**	**II**	**III**	**IV**	**L105X**	**Haplotype**					**PTT**	**Haplotype**	
					**D13S1694**	**D13S1695**	**D13S1697**	**D13S1698**	**D13S260**		**TAAA-Rpt**	**TTTA-Rpt**
I: 1				[Negative]	[**2**,2]	[**3**,3]	[**1**,3]	[**2**,2]	[**4**,5]	[Positive]	[**170**,186]	[292, **288**]
I: 2				[Positive]	[**2**,3]	[**3**,4]	[**2**,1]	[**3**,3]	[**5**,4]	[Negative]	[**178**,178]	[288, **292**]
												
	II: 1			Positive	**2**,3	**3**,4	**3**,1	**2**,3	**5**,4	Negative	**178**,186	**288**,292
												
	II: 3									x	[**?**,178]	[**?**,292]
	*II: 4*			x						x		
		III: 2		Negative						Negative	**178**,178	**292**,296
												
	II: 7			[Positive]	[**2**,3]	[**3**,4]	[**3**,1]	[**2**,3]	[**5**,4]	[Negative]	[**186**,178]	[**292**,292]
	*II: 8*			x	[**2**,2]	[**3**,3]	[**1**,1]	[**2**,3]	[**3**,4]	[Negative]	[**178**,178]	[**292**,288]
		III: 4		Positive	**2**,3	**3**,4	**1**,1	**2**,3	**3**,4	Negative	**178**,186	**292**,292
		III: 5		Positive	**2**,3	**3**,4	**1**,1	**3**,3	**4**,4	Negative	**178**,178	**288**,292
		III: 6		Negative	**2**,2	**3**,3	**1**,3	**3**,2	**4**,5	Negative	**178**,178	**288**,292
		III: 7		Positive	**2**,3	**3**,4	**1**,1	**3**,3	**4**,4	Negative	**178**,178	**292**,292
												
	II: 9											
	*II: 10*			Negative						[Negative]		
		III: 8		Negative						Negative	**178**,186	**288**, 296
		III: 9		Negative						Negative	**178/**178	**292/**292
												
	II: 11			[Negative]	[**x**,2]	[**x**,3]	[**x**,2]	[**x**,3]	[**x**,5]	[Positive]	[**170**,178]	[**288**,292]
	*II: 12*			Negative	**2**,2	**3**,3	**1**,2	**2**,2	**4**,4	Negative	**182**,190	**292**,284
		III: 10		Negative	**2**,2	**3**,3	**1**,2	**2**,3	**4**,5	Positive	**170**,190	**288**,284
		III: 11		Negative	**2**,2	**3**,3	**1**,2	**2**,3	**4**,5	Negative	**178**,182	**292**,292
												
	II: 13				[**2**,2]	[**3**,3]	[**1**,2]	[**2**,3]	[**4**,5]	[Positive]	[**170**,178]	[**288**,292]
	*II: 14*			Negative	**2**,2	**2**,4	**2**,2	**3**,2	**1**,2	Negative	**174**,190	**284**, 296
		III: 12		Negative	**2**,2	[**2**,3]	**2**,1	**3**,2	**1**,4	Negative	**174**,178	**296**,292
		III: 13		Negative	**2**,2	**3**,4	**2**,2	**3**,2	**5**,2	[Negative]	**174**,178	**296**,292
		III: 14		Negative	**2**,2	**3**,4	**2**,2	**3**,2	**5**,2	Negative	**174**,178	**296**,292
		III: 15		Negative	**2**,2	**3**,4	**1**,2	**2**,2	**4**,2	Positive	**170**,190	**288**,284
												
	II: 16			Positive	**2**,3	**3**,4	**1**,1	**2**,3	**4**,4	Negative	**178**,186	**288**,292
	*II: 15*			x						x	[**174**,?]	[**288**,?]
		III: 16		Negative						Negative	**174**,186	**288**,292
		III: 17		[Positive]		3,4		3,3	1,4	x		
		III: 18		Positive						x		
												
	II: 18			Negative	**2**,2	**3**,3	**1**,2	**2**,3	4,5	Positive	**170**,178	**288**,292
	*II: 17*			x						[Negative]	[**178**,178]	[**292**,288]
		III: 19		Negative						Positive	**170**,178	**288**,292
			IV: 1	Negative						Negative	**178**,178	**292**,292
			IV: 2	Negative						Negative	**178**,178	**292**,292
		III: 20		Negative						Negative	**178**,178	**288**,292
		III: 21		Negative						Negative	**178**,178	**288**,292
												
	II: 20			[Positive]	[**2**,3]	[**3**,4]	[**1**,1]	[**2**,3]	[**4**,4]	[Positive]	[**170**,178]	[**288**,292]
	*II: 21*			Negative	**1**,1	**1**,3	**1**,1	**1**,2	**1**,5	Negative	**178**,190	**284**,292
		III: 22		Positive	**1**,3	**1**,4	**1**,1	**1**,3	**1**,4	Negative	**178**,190	**292**,292
		III: 23		Negative	[**1**,2]	**1**,3	**1**,1	**1**,2	**1**,4	Negative	**178**,178	**284**,292
		III: 24		Negative	**1**,2	**1**,3	**1**,1	**1**,2	**1**,4	[Negative]	**178**,190	**292**,292
		III: 25		Positive	**1**,3	**1**,4	**1**,1	**1**,3	**1**,4	Positive	**170**,178	**288**,284

*Italic*: married to MON1080 family member. **Bold number:** distinguish the phase of the hapotypes. [Brackets and grey text]: result inferred (not from direct testing. II:10 -PTT not done, status determined by real-time PCR). Since all results regarding the parents are inferred, the *BRCA2* mutation was assigned arbitrarily to one individual from generation I. Linked haplotype: *BRCA2*: 3, 4, 1, 3, 4 *MSH2*: 170, 288.

). Primer sequences and PCR amplification conditions can be found in the Genome Database (GDB, http://gdbwww.gdb.org/gdb/). PCR products were separated on denaturing acrylamide gels and visualised by autoradiography.

## RESULTS

### Immunohistochemistry analysis

**Figure 2:**
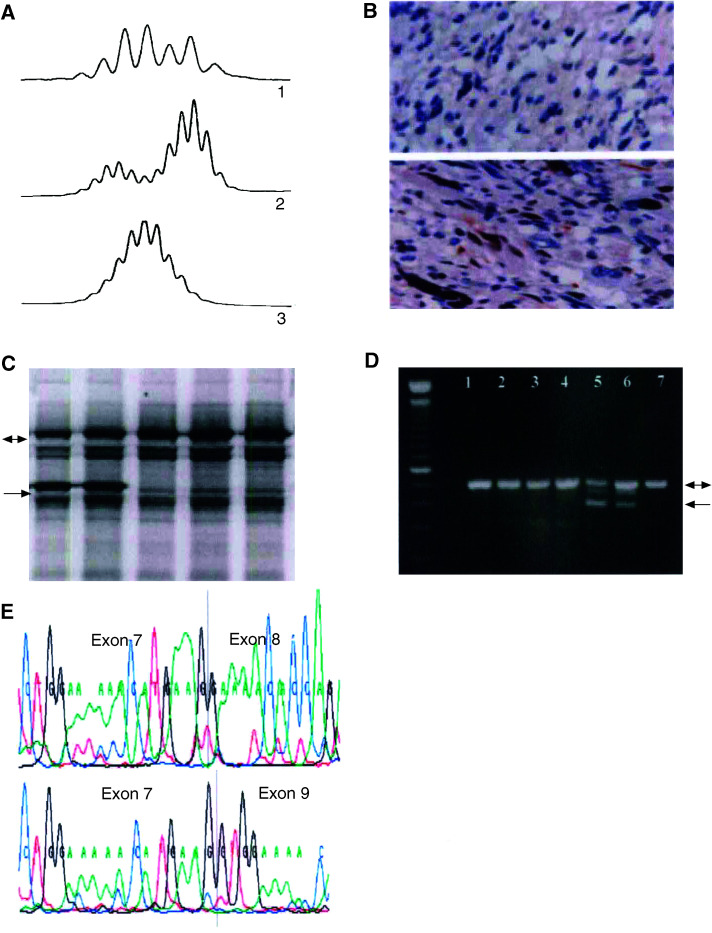
(**A**) Microsatellite instability at various loci in astrocytoma of patient II: 20. (1) D2S123; (2) BAT 25 and (3) BAT 26. (**B**) Immunohistochemical staining for MSH2 and MLH1 in the astrocytoma of patient II: 20. Upper panel: lack of staining for the MSH2 antibody indicating MSH2 inactivation in the tumour. Lower panel: normal staining of MLH1 in the astrocytoma (× 400). (**C**) Protein truncation test shows a truncated protein in patients II: 18 and III: 25. (Lanes 1 and 2) the double-headed arrow indicates the protein product from the normal allele. The single arrow indicates the truncated protein. (**D**) PCR products of cDNA segment including exons 5–9. Lanes 1–7: the double headed arrrow indicates PCR product of normal allele. Lanes 5 and 6: single arrow indicates PCR product of mutant allele. (**E**) The first sequence of cDNA shows the junction of exons 7 and 8. The second sequence shows the exon 8 deletion found in patients with HNPCC-related cancers in family MON1080.

Several pathology samples from patient III: 25 were evaluated by IHC. A moderately well differentiated, endometrioid adenocarcinoma of the uterus showed weak nuclear staining of the carcinoma with both antibodies. The second sample was a small villotubular adenoma that showed strong nuclear staining with the MLH1 antibody, whereas no staining with the MSH2 antibody was observed in the adenoma. Thus, there was no evidence of mismatch repair-deficiency from inactivation of MLH1 in either of these two samples, but the findings suggested *MSH2* inactivation in the colonic adenoma. Immunohistochemistry for MLH1 and MSH2 was also performed on the anal squamous cell carcinoma and keratoacanthoma from patient III: 10. Both lesions displayed nuclear staining with the two antibodies, showing no evidence of MLH1 or MSH2 inactivation in these samples. The results for the brain tumour from II: 11 suggested inactivation of MSH2 (Figure 2

), as shown by the lack of staining in the astrocytoma, whereas IHC staining with the MLH1 antibody was normal. Immunohistochemistry analysis of the available BCs is shown in Table 2

**Table 2:** Female breast cancer: pathological analysis and immunohistochemistry (IHC) results of selected markers in family MON1080

					**IHC**					
**Family ID**	***BRCA2*/*MSH2***	**Age at diagnosis**	**Vital status**	**Breast cancer histopathology**	**ER**	**PR**	**CDH-1**	**MIB-1**	**p53**	**HER2/ErbB-2**
III: 5	mut/wt	42	Alive	LCIS with six foci of infiltrating lobular carcinoma	+++	+++	−	+	++	−
III: 17	mut/wt	33	Deceased	DCIS with infiltrating ductal carcinoma (2.5 cm). Extensive lobular cancerisation	ND	ND	ND	ND	ND	−
III: 18	mut/wt	33	Deceased	Two foci (1.6 and 1 cm) of infiltrating lobular carcinoma	+++	+	+++	+	++	−
III: 22	mut/wt	34	Deceased	Multicentric infiltrating lobular carcinoma	+++	+++	−	−	+/−	−
III: 25	mut/mut	32	Alive	LCIS with infiltrating lobular carcinoma (1.5 cm). Separate foci of DCIS	+	+	ND	ND	ND	ND

Mut=heterozygous mutation; Wt=wild-type; LCIS=lobular carcinoma *in situ*; DCIS=ductal carcinoma *in situ*; ER=oestrogen receptor; PR=progesterone receptor; CDH-1=E-cadherin.

. In particular, III: 25, discussed above, developed an invasive BC, which was ER/PR positive. None of the four breast tumours analysed overexpressed HER2. Loss of E-cadherin expression was noted in two breast cancers, whereas in one case it was overabundant.

### MSI analysis

Of the five samples subjected to MSI analysis, one (III: 25; DNA from a villotubular adenoma) showed high degree of instability at three out of five loci (BAT25, BAT26, D5S346), and the endometrioid adenocarcinoma of the uterus was MSI-L (only BAT-25 was unstable). Interestingly, patient II: 11, from whom brain tumour tissue was available, showed MSI for BAT-25 and D2S123; the other loci were stable (Figure 2). The keratoacanthoma and the anal carcinoma from patient III: 10 were microsatellite stable (MSS).

### *MSH2* mutation analysis

A truncated protein in the *MSH2* gene was identified by PTT in one woman (III: 25) who had a colon polyp and endometrial cancer. To precisely locate the underlying cause of the truncation, we used cDNA to amplify a segment spanning exons 5–9. Electrophoretic separation of the PCR products showed the presence of an additional smaller fragment. Sequencing of this fragment revealed a deletion of exon 8 from the cDNA segment (Figure 2). The deletion is out of frame, and results in a stop codon 12 nucleotides downstream of exon 9 (at nucleotide 1398). DNA sequencing of the exon 8 and the flanking intronic regions did not reveal any alteration of the consensus splice sites, indicating that the exon 8 deletion was most likely caused by a large genomic deletion of the region encompassing exon 8.

In the quantitative real-time PCR analysis, normalised values for nondeleted patients and normal controls are equal to one; the normalised values for positive controls and deleted patients are equal to zero. Real-time PCR analysis confirmed the presence of a germline intragenic deletion encompassing exon 8 and flanking intronic sequences in the patients showing a truncation in MSH2 by PTT (Table 1). DNA from II: 20 was not available for testing, but the absence of the mutation in his widow's (data not shown) cDNA (II: 21) (as well as haplotype analysis, see below) were sufficient to confirm that II: 20, who died of CRC at 36 years of age, was an obligate carrier.

### *MSH2* genomic DNA sequencing

**Figure 3:**
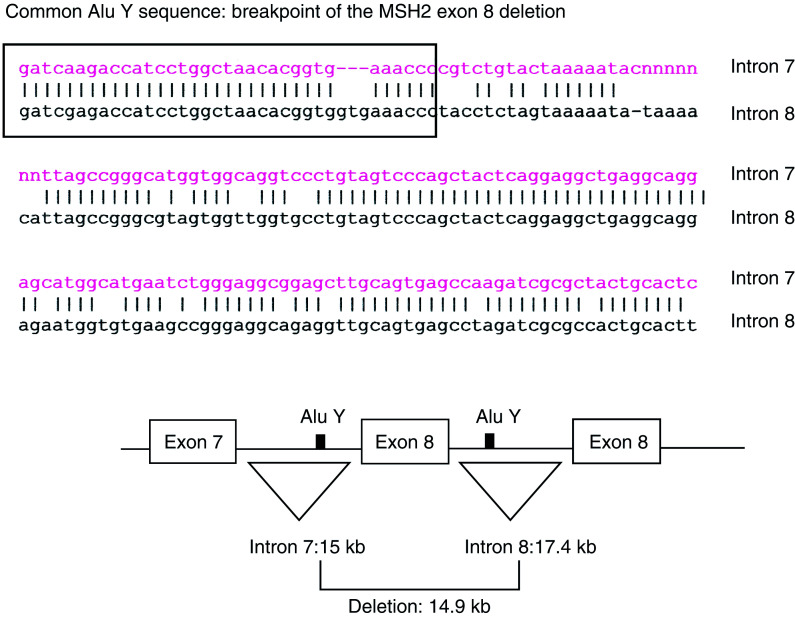
Characterisation of the *MSH2* exon 8 deletion: the intron 7 Alu Y shares 83% identity with the intron 8 Alu Y. There are 17 Alu family sequences in intron 7 and 18 Alu family sequences in intron 8. The MON1080 deletion is the result of the possible recombination between identical 45 bp sequences found in both AluY sequences.

Long-range PCR on genomic DNA was used to confirm the deletion and to locate the region containing the breakpoint. Different sets of primers were used to exactly characterise the deletion at the junction site. A 1.9 kb fragment containing the deletion breakpoint was then amplified from genomic DNA with the following primers: H2 EX8 ALUYF and MSH2, IVS8 10318R. The exon 8 deletion was identified as a 14.9 kb deletion occurring between two Alu sequences. The localisation of the breakpoint lies within a sequence of 45 bp that is identical in both Alu sequences that had been sequenced (Figure 3

).

### *BRCA2* mutation analysis

DNA sequencing performed by Myriad Genetic Laboratories on patient III: 22, who had lobular breast cancer, uncovered a *BRCA2* exon 3 542G>T mutation, predicted to result in a truncated protein (L105X). Patient III: 25 was shown to carry the same mutation by DNA sequencing. Subsequently, SSCA analysis was used to test all available family members for the 542G>T mutation. We confirmed that II: 20, the father of III: 22 and III: 25, is an obligate carrier of the *BRCA2* exon 3 542G>T mutation and an obligate double heterozygote because his widow (II: 21), the mother of III: 22 and III: 25, does not carry the *BRCA2* mutation or the *MSH2* mutation. Nine individuals were found to carry the L105X mutation: five of them are affected by BC and one was diagnosed with an ovarian papillary serous adenocarcinoma. Three mutation carriers are not affected, II: 1 (a 74 years-old male), III: 4 (a 53-year-old female) and III: 7 (a 42-year-old female) (Figure 1 and Table 1).

### Haplotype analysis

Haplotype analysis using polymorphic markers spanning *MSH2* and *BRCA2* was performed on 26 individuals. The *MSH2* linkage results were based on two markers, while the haplotype associated with the *BRCA2* exon 3 542G>T mutation was assessed using five markers (Table 1). For both *MSH2* and *BRCA2*, we identified a common haplotype shared by all mutation carriers, consistent with the inferred common ancestry. The five *MSH2* exon 8 deletion carriers (III:10, III:15, II:18, III:19 and III:25, Table 1) shared an allele of identical size for two different *MSH2* markers: TTTA-Repeat (intron 2) and TAAA-Repeat (intron 8) (alleles 170 and 288). All carriers of the cDNA deletion of the exon 8 are heterozygous for these two markers. A further four individuals were classified as obligate mutation carriers on the basis of reconstructed haplotypes and/or their position in the pedigree. We used somatic cell hybrids containing a maternally derived or a paternally derived chromosome 2 obtained from GMP Genetics (Waltham, MA, USA) from individual II: 18 to confirm the phase of the haplotype.

By direct genotyping, eight individuals in the MON1080 family (II: 2, III: 4, III: 5, III: 7, II: 16, III: 18, III: 22, III: 25) were found to share a common allele for five different *BRCA2* markers (3, 4, 1, 3, 4). In seven of eight cases, we were able to rule out double heterozygosity for the exon 8 deletion. Patient III: 17 was only tested for three markers, but the result is consistent with the presence of a *BRCA2* mutation and she was diagnosed with breast cancer at age 33 years. She is not a carrier of the *MSH2* mutation. In contrast, her cousin, III: 25, is a double heterozygote, and her father is an obligate double heterozygote (Table 1). Three other individuals are obligate mutation carriers.

## DISCUSSION

We initially investigated the MON1080 family because of concern regarding BC risk, but examination of the pedigree showed that the incidence of CRC was just as high as that of BC (Figure 1). This family seemed an appropriate kindred in which to look for a novel breast–CRC predisposition gene. Linkage and molecular genetic analyses, however, revealed two independent mutations: a mutation in one MMR gene (*MSH2*) and in one BC susceptibility gene (*BRCA2*). There is no evidence that women in this pedigree were susceptible to BC solely because they carried an MMR gene mutation, or that CRC was associated with *BRCA2* mutations, as has been previously suggested (Risch *et al*, 2001). Two individuals (II: 20 and his daughter III: 25) are double heterozygotes. The *BRCA2* exon 3 542G>T mutation was present in nine family members, among whom five women were diagnosed with BC and one was diagnosed with ovarian cancer. Three carriers are unaffected; two women had preventive mastectomies, and the third was a male, who recently died of a noncancer-related condition. In addition, eight *MSH2* mutation carriers were identified in the MON1080 family. Mutation status was inferred for three individuals: two died of CRC and one died of an astrocytoma following a rectal cancer. Three additional individuals are affected by invasive cancer, while two remain unaffected by an HNPCC-related cancer. However, among the unaffected individuals, one has had two colon polyps removed that were not available for IHC or MSI testing and the other had both an anal squamous cell carcinoma and a keratoacanthoma that were MSS and did not show loss of MSH2 by IHC. The family history did not reveal additional cases of keratoacanthoma or other skin cancers. In patients with sebaceous gland adenocarcinoma (SGC) and a personal or family history of CRC, the diagnosis of MTS is clear. However, lack of a positive family history of SGC, absence of MSI and normal expression of *MSH2* in tumours of patients indicate that the skin tumour can be considered a sporadic SGC (Entius *et al*, 2000). In this case, we suspect the keratoacanthoma is a sporadic occurrence and therefore a diagnosis of MTS is not sustained.

The histopathology of the breast tumours in the *BRCA2* exon 3 542G>T mutation carriers is interesting (Table 2). Of the five female mutation carriers affected with breast cancer, one had a ductal carcinoma *in situ*, with foci of microinvasion, but the other four affected women had lobular invasive BC. Two of these four had areas of lobular carcinoma *in situ* as well. Our finding of four cases of lobular breast cancer in this family is consistent with results from our previous hospital-based series of breast cancers from Montreal, where we identified four *BRCA2* mutation carriers among 127 women with first primary invasive BCs (Chappuis *et al*, 2001). Two of the four *BRCA2* mutation carriers had lobular BC, and lobular BCs were statistically significantly overrepresented in *BRCA2* mutation carriers compared with noncarriers. Invasive lobular BC is seen in 4% of *BRCA1*-related breast cancer and 4% of sporadic controls, whereas 11% of *BRCA2*-related breast cancer is of this subtype (Chappuis *et al*, 2000). Taken together, these findings suggest that lobular BC is more commonly a feature of germline *BRCA2* mutation carriers than it is of *BRCA1* mutation carriers, and its presence could be an indication to commence molecular studies of *BRCA1/2* with *BRCA2* analysis.

MSI-H and absence of *MSH2* protein were observed in an astrocytoma removed from II: 11 (Figure 2). This patient is an obligate carrier of the *MSH2* exon 8 deletion since his son carries this mutation. Leung *et al* previously reported the presence of a germline MMR gene mutation and MSI in patients with sporadic gliomas. They analysed four patients with microsatellite-unstable gliomas. Three had loss of *MSH2* protein by IHC and carried a germline *MSH2* mutation. The fourth individual had a germline *MLH1* mutation. Interestingly, only one of them reported a family history of CRC (Leung *et al*, 1998). Vasen *et al* (1996) suggested that the possible histological types of brain tumours that can occur in HNPCC kindreds include astrocytomas, oligodendrogliomas and rarely ependyomas. Other studies have reported germline MMR gene mutation in four patients with Turcot syndrome; three reported that a close relative had had CRC (Vasen *et al*, 1996; Miyaki *et al*, 1997; Hamilton *et al*, 2002).

Carriers of the *MSH2* exon 8 deletion in this family share an identical disease haplotype (Table 1). The haplotype of the grandparents of the mutation carriers was determined by inference and confirmed the consistency of our haplotype analysis. The frequency of the haplotype of this *MSH2* mutation in the general population is not yet known, and it will be interesting to establish if other kindreds with exon 8 deletions have the same haplotype flanking *MSH2* as reported here. Quantitative real-time PCR analysis confirmed that the germline intragenic deletion encompasses exon 8 and flanking intronic sequences. It is present in all patients showing a truncated protein by PTT for whom DNA was available for testing. Long-range PCR confirmed the exon 8 deletion, by revealing a 1.9 kb fragment containing the deletion breakpoint. The 14.9 kb deletion is the likely result of recombination between identical 45 bp sequences found in both AluY sequences (Figure 3). Wang *et al* characterised several large genomic deletions in *MSH2* (exons 11–14; exons 12–15) and *MLH1* (exon 4; exons 7–10) in patients suspected of HNPCC. They sequenced five deletion breakpoints where Alu-repetitive elements may be involved, but Alu-mediated recombination was not clearly demonstrated. Interestingly, they detected a deletion of exon 8 in the *MSH2* gene in a patient by semiquantitative multiplex PCR, but because of the large intronic sequence they could not characterise the deletion breakpoint by long-rang PCR (Wang *et al*, 2003). By contrast, in the kindred reported here, following the observation of heterozygosity for intragenic *MSH2* markers TAAA-Rpt (Table 1) indicating that the deletion breakpoint would be localised downstream from this repetitive element, we were able to definitely characterise this mutation (Figure 3). It seems highly likely that other exon 8 cDNA deletions reported in the literature are due to inter-Alu recombination as this region contains a large number of Alu-repetitive elements (17 Alu family sequences in intron 7; 19 Alu family sequences in intron 8), which could promote such deletions. The frequency of such large genomic deletion is probably underestimated; the PCR-based methods used in most molecular genetics laboratories do not allow their detection. In fact, Charbonnier *et al* (2002) estimated that large MMR gene rearrangements account for about 10% of the HNPCC cases in France. Wijnen *et al* (1998) reported similar findings in the Dutch population; 6.5% of the HNPCC cases are due to intragenic deletion in *MLH1* or *MSH2*. Genomic rearrangements are an important component of the MMR mutation spectrum, and Alu-mediated recombination are likely responsible of a large amount of these intragenic deletions (Nystrom-Lahti *et al*, 1995; Viel *et al*, 2002).

In this large BC/CRC kindred, we identified disease-associated mutations in both *BRCA2* and *MSH2*. This is the first report of double heterozygotes with truncating mutations in predisposition genes for BC and CRC. The presence of the *BRCA2* mutation explained the occurrence of breast and ovarian cancer in the kindred, and presence of the *MSH2* mutation accounted for CRC, endometrial and brain cancer (astrocytoma). One individual with nonmedullary thyroid cancer (and breast cancer) carried a *BRCA2* mutation. There were also two individuals with lung cancer, one of whom (II:7) is known to carry the *BRCA2* mutation on the basis of the reconstructed haplotype and the presence of this mutation in his children. By reconstruction, he does not carry the *MSH2* mutation. We could not establish the mutation status of the other male subject with lung cancer (II:9) at either locus (Figure 1 and Table 1).

Our results support the notion that early-onset BC in HNPCC may be due to mutations in other genes, such as *BRCA2*, and that early-onset CRC in *BRCA1/2* may be due to mutations in MMR genes, such as *MSH2*. This report describes the phenotype of the first two individuals in whom truncating mutations in both genes have been identified. Clearly, it is not possible to draw firm conclusions from only two cases, but the double heterozygotes in this kindred do not appear to have an earlier age of onset than carriers of a single mutation, and the cancers appear to have independent genetic aetiologies. Moreover, based on the data presented here and other recent results (Muller *et al*, 2002), we do not consider that a single highly penetrant BC–CCR cancer susceptibility gene is likely to exist. Rather, in agreement with the most recent epidemiological reports, our findings argue that the two conditions (CRC and BC) are independent of each other (Newschaffer *et al*, 2001). Kindreds with the phenotype reported here are not frequently identified, but this detailed report demonstrates how a single family can contribute more generally to the understanding of the development of cancer in unusual situations such as double heterozygosity.
